# Supplementary material for: Identification of differentially recognized T cell epitopes in the spectrum of tuberculosis infection
Source: Nat Commun. 2024 Jan 26;15:765. doi: 10.1038/s41467-024-45058-9 (PMC10817963; doi:10.1038/s41467-024-45058-9)
Supplement: Supplementary file 1 — Supplementary Information [file 41467_2024_45058_MOESM1_ESM.pdf]

SUPPLEMENTARY Figures and Tables

Figure S1

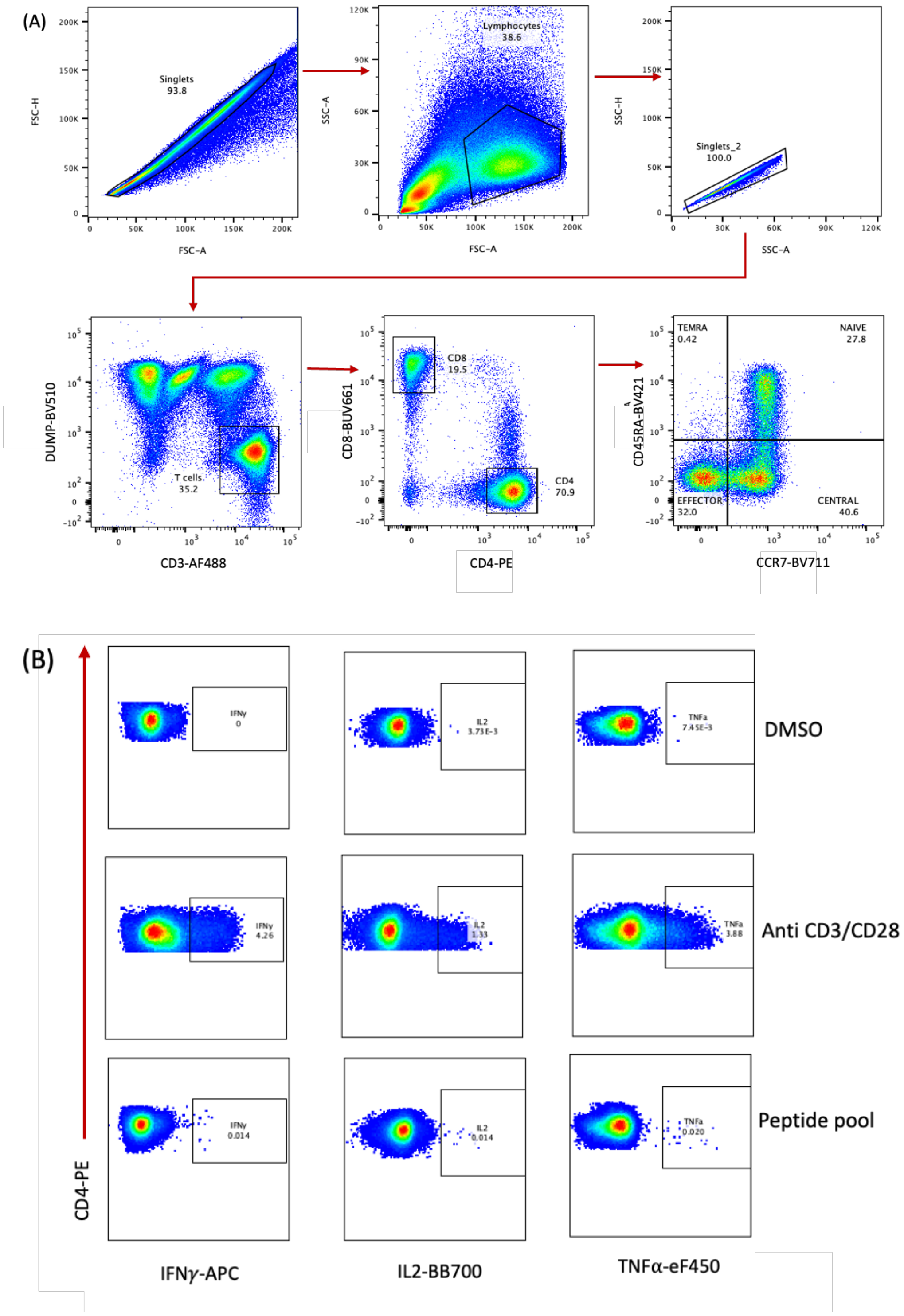

Figure S1: Gating strategy for flow cytometry experiments. (A) A representative strategy used to define cytokine-producing T cells. (A)PBMCs were stained and gated on singlets (FSC-A/FSC-H), lymphocytes, singlets (SSC-A/SSC-H), live CD19- CD14- CD3+ T cells, and CD4+ or CD8+ T cells. These were gated on memory populations based on CD45RA and CCR7

expression. (B) Definition of cytokine-producing CD4 T cells stimulated with DMSO (negative control: top), anti-CD3/CD28 (positive control: middle), and peptide pool (MTB300: bottom).

Table S1. Antibodies used for flow cytometry experiments

Marker	Clone	RRID	Fluorochrome	Manufacturer	Volume per one million cells
CD3	UCHT1	AB_389310	AF488	Biolegend	2ul
CD4	RPA-T4	AB_395752	PE	BD biosciences	1ul
CD8	RPA-T8	AB_2874820	BUV661	BD biosciences	1ul
CD45RA	HI-100	AB_10965547	BV421	Biolegend	2ul
CD19	HIB19	AB_2561668	BV510	Biolegend	1ul
CD16	3G8	AB_2562085	BV510	Biolegend	1ul
CD20	2H7	AB_2561941	BV510	Biolegend	1ul
CD14	63D3	AB_2716229	BV510	Biolegend	1ul
CCR4	1G1	AB_396907	PE-Cy7	BD biosciences	1.5ul
CCR7	G043H7	AB_2563865	BV711	Biolegend	1.5ul
CCR6	11A9	AB_2833076	BUV496	BD biosciences	1ul
CXCR3	G025H7	AB_2563157	BV605	Biolegend	1ul
TNF-alpha	MAb11	AB_2043889	eF450	Invitrogen	1.5ul
IFN-gamma	4S.B3	AB_469506	APC	Invitrogen	1.5ul
IL-2	MQ1-17H12	AB_2744488	BB700	BD biosciences	2.5ul
Live/Dead	65086614		eF506	Invitrogen	0.2ul
