# Supplementary material for: Identification of differentially recognized T cell epitopes in the spectrum of tuberculosis infection
Source: Nat Commun. 2024 Jan 26;15:765. doi: 10.1038/s41467-024-45058-9 (PMC10817963; doi:10.1038/s41467-024-45058-9)
Supplement: Supplementary file 3 — Description of Additional Supplementary Files [file 41467_2024_45058_MOESM3_ESM.pdf]

Description of Additional Supplementary Files

File Name: Supplementary Data 1
Description: T cell epitopes recognized by 21 participants with ATB (mid-treatment)
