# Supplementary material for: Identification of differentially recognized T cell epitopes in the spectrum of tuberculosis infection
Source: Nat Commun. 2024 Jan 26;15:765. doi: 10.1038/s41467-024-45058-9 (PMC10817963; doi:10.1038/s41467-024-45058-9)
Supplement: Supplementary file 5 — Reporting Summary [file 41467_2024_45058_MOESM5_ESM.pdf]

nature portfolio

Corresponding author(s): Dr. Cecilia Lindestam Arlehamn

Last updated by author(s): Dec 21, 2023

Reporting Summary

Nature Portfolio wishes to improve the reproducibility of the work that we publish. This form provides structure for consistency and transparency in reporting. For further information on Nature Portfolio policies, see our Editorial Policies and the Editorial Policy Checklist.

Statistics

For all statistical analyses, confirm that the following items are present in the figure legend, table legend, main text, or Methods section.

n/a	Confirmed	
☐	☒	The exact sample size (*n*) for each experimental group/condition, given as a discrete number and unit of measurement
☐	☒	A statement on whether measurements were taken from distinct samples or whether the same sample was measured repeatedly
☐	☒	The statistical test(s) used AND whether they are one- or two-sided<br>*Only common tests should be described solely by name; describe more complex techniques in the Methods section.*
☒	☐	A description of all covariates tested
☐	☒	A description of any assumptions or corrections, such as tests of normality and adjustment for multiple comparisons
☐	☒	A full description of the statistical parameters including central tendency (e.g. means) or other basic estimates (e.g. regression coefficient) AND variation (e.g. standard deviation) or associated estimates of uncertainty (e.g. confidence intervals)
☐	☒	For null hypothesis testing, the test statistic (e.g. *F*, *t*, *r*) with confidence intervals, effect sizes, degrees of freedom and *P* value noted<br>*Give P values as exact values whenever suitable.*
☒	☐	For Bayesian analysis, information on the choice of priors and Markov chain Monte Carlo settings
☒	☐	For hierarchical and complex designs, identification of the appropriate level for tests and full reporting of outcomes
☒	☐	Estimates of effect sizes (e.g. Cohen's *d*, Pearson's *r*), indicating how they were calculated

Our web collection on statistics for biologists contains articles on many of the points above.

Software and code

Policy information about availability of computer code

Data collection	Mabtech Apex version 1.1.59.128 for fluorospot data, Mycobrowser tool for Mtb protein information
Data analysis	Data analyses were performed using FLow Jo V10 (for flow analysis) and GraphPad Prism version 9.2 software (for statistical analysis).

For manuscripts utilizing custom algorithms or software that are central to the research but not yet described in published literature, software must be made available to editors and reviewers. We strongly encourage code deposition in a community repository (e.g. GitHub). See the Nature Portfolio guidelines for submitting code & software for further information.

Data

Policy information about availability of data

All manuscripts must include a data availability statement. This statement should provide the following information, where applicable:

- Accession codes, unique identifiers, or web links for publicly available datasets
- A description of any restrictions on data availability
- For clinical datasets or third party data, please ensure that the statement adheres to our policy

The identified epitopes from the proteome-wide screen in participants with ATB has been submitted to IEDB (Immune Epitope Database) which is a publicly accessible repository. It can be found under the submission ID: 1000914 (https://www.iedb.org/result_v3.php?cookie_id=41916e). H37Rv genome was used as reference genome (genebank accession number NC_000962) for comparing proteins. All data required to state the conclusions in the paper are present in the paper and/or the supplementary data.

Research involving human participants, their data, or biological material

Policy information about studies with human participants or human data. See also policy information about sex, gender (identity/presentation), and sexual orientation and race, ethnicity and racism.

Reporting on sex and gender	Sex or Gender were not considered in the study design. However, we did not use single gender cohort. From Peru, 21 participants with ATB who were mid-treatment (3-4 months post diagnosis) were recruited from 2012 to 2013. The cohort consisted of 62% males and 38% females. From Sri Lanka, a cohort of patients with ATB (at diagnosis, n=24; 96% males and 4% females), IGRA+ individuals (n=25; 48% males and 52% females), and IGRA- individuals (n=43; 39% males and 60.5% females) were recruited from 2019-2022. For the Moldova cohort, biological sex or gender was not captured.
Reporting on race, ethnicity, or other socially relevant groupings	In our study, we used study participants from Peru for the proteome-wide screening. We extended the study participant cohort to include individuals from Sri Lanka and Moldova for validation experiments. No socially relevant categorization variables were used in the study.
Population characteristics	In the present study, all the study participants were in the age group of 18-50 years. Among the recruited anti-tuberculosis (ATB) patients, the focus was specifically on those with drug-sensitive pulmonary tuberculosis. This allowed us to minimize the effect of confounding factors related to different disease types, such as extrapulmonary TB or drug-resistant TB. Individuals with MDR or XDR-TB, and those diagnosed HIV, HBV or HCV infection were excluded, as well as patients with significant systemic diseases, including, for example, diabetes, renal disease, liver disease, uncontrolled hypertension, and malignancy.<br>For proteome wide screening, individuals with mid-treatment active pulmonary TB were enlisted from Peru, comprising 62% male and 38% female participants. To validate the response to the peptide pool, we extended the study participant cohort to include individuals from Sri Lanka and Moldova.
Recruitment	After obtaining ethics approval and informed consent, participants aged 18-50 were recruited based upon the inclusion and exclusion criteria. Individuals with ATB had symptomatic pulmonary TB, diagnosed by a positive GenXpert (Cepheid, Inc.), positive sputum smear, and/or a positive culture. Individuals with MDR or XDR-TB, and those diagnosed HIV, HBV or HCV infection were excluded, as well as patients with significant systemic diseases, including, for example, diabetes, renal disease, liver disease, uncontrolled hypertension, and malignancy.<br>From Peru, 21 participants with ATB who were mid-treatment (3-4 months post diagnosis) were recruited from 2012 to 2013. They responded well to treatment and had regained any weight lost due to the infection. From Sri Lanka, a cohort of patients with ATB (at diagnosis, n=24), IGRA+ individuals (n=25), and IGRA- individuals (n=43) were recruited from 2019-2022. A subset of the patients with ATB (n=7) was followed longitudinally from the time of diagnosis until the end of treatment. They provided blood samples at diagnosis, 2 months post-diagnosis, and 6 months post-diagnosis. From Moldova, a cohort of patients with ATB (at diagnosis, n=9), ATB (mid-treatment, n=12), IGRA+ individuals (n=52), and IGRA- individuals (n=26) were recruited from 2018-2022. The IGRA+ and IGRA- individuals were household contacts of a patient with active TB (e.g., an "index case"). They provided blood samples up to 6 months after the index case received their ATB diagnosis.<br>Healthy participants were classified into IGRA+ (i.e., Latent TB infection) and IGRA- groups based on IGRA tests (QuantiFERON-TB Gold Plus, Cellestis and/or T-spot.TB, Oxford Immunotec). There were no selection bias which could impact the result.<br>Participant compensation:<br>Peru: Subjects received local supermarket vouchers that are equivalent of 60 nuevos soles ($21.40 USD, 1USD = 2.80 nuevos soles) for the initial blood draw done at screening. Subjects who underwent leukapheresis received an additional voucher of 300 nuevos soles ($107 USD) in addition to a light meal at the leukapheresis and transportation cost for the visits.<br>Sri Lanka: Participants were provided with Rs 1000 for travel, a voucher worth Rs.2000 from a supermarket to buy food items and HbA1c testing free of charge.<br>Moldova: Participant compensation were equivalent of US$10 per patient per visit, which is an amount that is customary and allowable by local norms, as well as Moldova IRB requirements.
Ethics oversight	Ethical approval was obtained from the institutional review boards at La Jolla Institute for Immunology (LJI; Protocol Numbers: VD-090, VD-143, VD-175), Universidad Peruana Cayetano Heredia (66754), Phthisiopneumology Institute (CE-3/2018), University of California San Diego (180068), and University of Colombo for General Sir John Kotelawala Defense University, Sri Lanka (EC18-122, EC15-094).

Note that full information on the approval of the study protocol must also be provided in the manuscript.

Field-specific reporting

Please select the one below that is the best fit for your research. If you are not sure, read the appropriate sections before making your selection.

- [x] Life sciences
- [ ] Behavioural & social sciences
- [ ] Ecological, evolutionary & environmental sciences

For a reference copy of the document with all sections, see nature.com/documents/nr-reporting-summary-flat.pdf

Life sciences study design

All studies must disclose on these points even when the disclosure is negative.

Sample size	In this descriptive observational study, no sample calculation was performed. The samples were recruited from three different geographical sites, UPCH in Peru, the General Sir John Kotelawala Defense University in Sri Lanka, and the Phthisiopneumology Institute in the Republic of Moldova. As many participants as possible were used for the experiment
Data exclusions	No data were excluded from the analyses
Replication	The peptide and pool stimulation conditions in the fluorospot experiments were performed in triplicates. The average of SFC values were used in the graphs. Each pool or peptide was considered positive compared to the background that had a equivalent amount of DMSO based on the following criteria: i) 20 or more spot-forming cells (SFC) per million PBMC after background subtraction, ii) a greater than 2-fold increase compared to the background, and iii) $p<0.05$ by student's t-test or Poisson distribution test when comparing the peptide or pool triplicates with the negative control. Positive pool responses were replicated during the deconvolution experiments.<br>For flow cytometry experiments, samples with enough cells were run in duplicates. All attempts at replication were successful.
Randomization	The study participants used in this study were randomly allocated to each experimental group. All individuals in the analysed study cohorts were categorized using predefined classification criteria, and all available samples from these individuals were analysed as described. ATB individuals were the study subjects who had clinical signs and symptoms of pulmonary TB (confirmed by the physician). IGRA+ were the individuals who tested positive for IGRA test (interferon gamma release assay). IGRA- individuals were negative for IGRA test. BOTH IGRA+ and IGRA- individuals did not exhibit any clinical signs or symptoms of tuberculosis.
Blinding	No blinding for experiments with a single cohort of participants. Individual running the experiments with multiple cohorts were blinded during set up and unblinded at the analysis stage.

Reporting for specific materials, systems and methods

We require information from authors about some types of materials, experimental systems and methods used in many studies. Here, indicate whether each material, system or method listed is relevant to your study. If you are not sure if a list item applies to your research, read the appropriate section before selecting a response.

Materials & experimental systems

n/a	Involved in the study	
☐	☒	Antibodies
☒	☐	Eukaryotic cell lines
☒	☐	Palaeontology and archaeology
☒	☐	Animals and other organisms
☐	☒	Clinical data
☒	☐	Dual use research of concern
☒	☐	Plants

Methods

n/a	Involved in the study	
☒	☐	ChIP-seq
☐	☒	Flow cytometry
☒	☐	MRI-based neuroimaging

Antibodies

Antibodies used

CD3-AF488, UCHT1 AB_389310 (Biolegend)
CD4-PE, RPA-T4 AB_395752 (BD biosciences)
CD8-BUV661, RPA-T8 AB_2874820 (BD biosciences)
CD45RA-BV421, HI-100 AB_10965547 (Biolegend)
CD19-BV510, HIB19 AB_2561668 (Biolegend)
CD16-BV510, 3G8 AB_2562085 (Biolegend)
CD20-BV510, 2H7 AB_2561941 (Biolegend)
CD14-BV510, 63D3 AB_2716229 (Biolegend)
CCR4-PE-Cy7, 1G1 AB_396907 (BD biosciences)
CCR7-BV711, G043H7 AB_2563865 (Biolegend)
CCR6-BUV496, 11A9 AB_2833076 (BD biosciences)
CXCR3-BV605, G025H7 AB_2563157 (Biolegend)
TNFalpha-alph-eF450, MAb11 AB_2043889 (Invitrogen)
IFN-gamma-APC, 4S.B3 AB_469506, (Invitrogen)
IL-2-BB700, MQ1-17H12 AB_2744488, (BD biosciences)
Live/Dead-eF506, 65086614, (Invitrogen)

Mabtech antibodies:
Mouse anti human ifng clone 1-D1K
mouse anti human Il-17 clone MT44.6

	anti-IFNg 7-B6-1-FS-BAM<br>anti IL-17 MT504-WASP<br>anti-BAM-490<br>anti-WASP-640
Validation	All the antibodies used in the study are validated and used by researchers (cited in the website of the company). We have titrated the amount required for each antibody in our experiments

Clinical data

Policy information about clinical studies

All manuscripts should comply with the ICMJEguidelines for publication of clinical research and a completedCONSORT checklist must be included with all submissions.

Clinical trial registration	Not applicable
Study protocol	The present study does not include any clinical trial
Data collection	The study subjects were recruited from three different cohorts. The samples were shipped from recruitment sites and stored in liquid nitrogen in the lab where the experiments were performed.<br>Peru: 2012-2013; Srilanka: 2015-2020; Moldova: 2018-2022
Outcomes	Not applicable

Flow Cytometry

Plots

Confirm that:

- [x] The axis labels state the marker and fluorochrome used (e.g. CD4-FITC).
- [x] The axis scales are clearly visible. Include numbers along axes only for bottom left plot of group (a 'group' is an analysis of identical markers).
- [x] All plots are contour plots with outliers or pseudocolor plots.
- [x] A numerical value for number of cells or percentage (with statistics) is provided.

Methodology

Sample preparation	The study utilized blood samples from which peripheral blood mononuclear cells (PBMCs) were isolated. PBMCs were obtained by density gradient centrifugation (Ficoll-Hypaque, Amersham Biosciences) from leukapheresis or whole blood samples, according to the manufacturer's instructions. The PBMC processing at the site in the Republic of Moldova used SepMate tubes (StemCell). Cells were resuspended in FBS (Gemini Bio-Products) containing 10% DMSO (v/v, Sigma) and cryopreserved in liquid nitrogen.<br>Cryopreserved PBMC were quickly thawed by incubating each cryovial at 37°C for 2 min, and cells transferred to cold medium (RPMI 1640 with L-glutamin and 25mM HEPES; Omega Scientific), supplemented with 5% human AB serum (GemCell), 1% penicillin streptomycin (Life Technologies), 1% glutamax (Life Technologies) and 20U/ml benzonase nuclease (MilliporeSigma). Cells were centrifuged and resuspended in complete RPMI medium to determine cell concentration and viability using trypan blue.
Instrument	Fluorospot analysis was done using IRIS Fluorospot reader, Mabtech, Sweden. FLow data was acquired using ZE5 cell analyzer (BioRad).
Software	FLowJo version 10 software. GraphPad Prism software (GraphPad Software, Inc., San Diego, CA, USA, version 9.2).
Cell population abundance	No cell sorting was performed in the present study
Gating strategy	Doublets were first excluded using FSC-A and FSC-H. Gating was then done on lymphocytes by FSC-A and SSc-A, followed by another singlet gate by FSC-A vs FSC-H. Then Total T cells were gated as CD3+ and negative for DUMP (L/D, B cells, monocytes) followed by CD8 or CD4 T cells. Each CD4 T cell subset was then evaluated for cytokine (IFN gamma, IL-2 and TNF alpha). For memory subsets, CD4 T cells were gated on CD45RA and CCR7 to get CCR7+CD45RA- (central memory), CCR7-CD45RA- (effector memory), CCR7+CD45RA+ (naïve), and CCR7-CD45RA+ (TEMRA).

- [x] Tick this box to confirm that a figure exemplifying the gating strategy is provided in the Supplementary Information.
